# Mate recognition in orchid bees is mediated by acquired chemical signals

**DOI:** 10.1093/beheco/arag124

**Published:** 2026-10-07

**Authors:** Marissa C Sandoval, Janosch Dohrs, Thomas Chouvenc, Thomas Eltz, Santiago R Ramírez

**Affiliations:** Department of Evolution and Ecology, University of California, Davis, Davis, CA 95616, USA; Department of Animal Ecology, Evolution and Biodiversity, Ruhr-Universität Bochum, 44801 Bochum, Germany; Department of Entomology and Nematology, University of Florida, Institute of Food and Agricultural Sciences, Ft. Lauderdale Research and Education Center, Davie, FL 33314, USA; Department of Animal Ecology, Evolution and Biodiversity, Ruhr-Universität Bochum, 44801 Bochum, Germany; Department of Evolution and Ecology, University of California, Davis, Davis, CA 95616, USA

**Keywords:** mate choice, courtship behavior, chemical signals, extended phenotype, Euglossine bees

## Abstract

The evolution of prezygotic reproductive barriers plays a central role in speciation and is often mediated by divergence in mating signals. Male orchid bees collect volatile compounds from the environment to concoct species-specific perfumes that function as sexual chemical signals during courtship display. While perfumes are thought to convey species recognition, their direct role in assortative mating remains unresolved. Here, we experimentally tested whether perfume composition facilitates mate recognition between 2 recently diverged and sympatric sister species, *Euglossa dilemma* and *E. viridissima*. We collected perfumes from males in sympatric regions of Mexico and experimentally supplemented *E. dilemma* males with either conspecific (*E. dilemma*) or heterospecific (*E. viridissima*) perfumes. We conducted single-choice mating assays and found that the rate of male courtship display did not differ between treatments. However, females of *E. dilemma* exhibited a strong preference for males with conspecific perfumes. While 77% of males loaded with *E. dilemma* perfume mated, only 8% of males loaded with *E. viridissima* perfume mated. Our results support the hypothesis that perfume composition alone can mediate reproductive isolation in orchid bees. These findings highlight the role of environmentally acquired signals as extended phenotypes that can rapidly generate behavioral isolation and contribute to speciation.

## Teaser

Naturalists have long been captivated by the courtship behavior of orchid bees. Male bees craft a perfume from environmental sources to attract females. Here, we demonstrate that perfumes function as a species recognition signal; we show that female bees prefer to mate with males that have supplemented their own species’ perfume over males with a related species' perfume. We think the differentiation in perfume has played a role in the diversification of orchid bees in the Neotropics.

## Introduction

The evolution of prezygotic reproductive barriers is considered the primary cause of speciation across diverse animal and plant taxa (Coyne et al. 1994). Prezygotic barriers can profoundly reduce gene flow between lineages, ultimately leading to complete reproductive isolation and speciation (Coyne and Orr 2004). Prezygotic barriers may arise from divergence of traits involved in courtship and mating behavior (*ie*, secondary sexual traits), including variation in behavior, phenology, physiology and morphology. Mating signals facilitate both recognition of conspecific mates as well as choice among mates by members of the opposite sex, and both processes can lead to directional selection on signal traits (Fisher 1930; Lande 1980, 1981; Kirkpatrick 1982).

Mating signals can vary among individuals and populations, and the observed variance of a signal trait depends on the strength of selection acting on it as well as constraints due to physiology, development and/or the underlying genetic architecture (Zuk et al. 1993; Gerhardt et al. 2000; Verzijden et al. 2005; Ballentine 2006; Bernal et al. 2006; Green et al. 2012; Selz et al. 2014; Leavell et al. 2022; Aubier et al. 2023). In some cases, mating signals may include features constructed from the environment that further increase the range of trait variability, *eg*, extended phenotypes (Schaedelin and Taborsky 2009). For instance, male bowerbirds gather environment-derived decorations in their display arena as part of their multifaceted courtship display, which also includes dances and complex behaviors (Endler et al. 2005; Endler 2012). Another example includes female stickleback fish that exhibit a strong preference for male fish that build shallow nests, with nest depth being a stronger predictor of mating success than intrinsic traits (*eg*, size or color) (Bolnick et al. 2015). However, due to the difficulties of manipulating traits and observing relevant behaviors in nonmodel organisms, few studies have directly tested the role of environmentally acquired signals in mate choice.

Among sensory modalities, chemoreception can mediate the comparatively rapid evolution of reproductive barriers because novel chemical communication systems can evolve at relatively low cost, with even single-receptor gene modifications producing variation in mate choice (Rosenthal 2017; Roitberg 2018). For this reason, insect sex pheromones provide a compelling way to explore behavioral questions regarding mate choice. The diversity of insect sex pheromones includes signals that are endogenously produced, sequestered from dietary sources, and, in exceptional cases, created from exogenous compounds directly (*ie*, pheromone analogs). Classic examples of endogenously synthesized pheromones have been investigated in moths, where often females advertise to receiving males and slight chemical changes can influence attraction (Butenandt et al. 1961; Bjostad et al. 1980; Kárpáti et al. 2008; Fujii et al. 2011; Sakurai et al. 2011). Further examples of volatile endogenous pheromones include scent-marking performed by male bumble bees (Frank 1941; Haas 1949; Bergman and Bergström 1997) and compounds produced by lekking male sand flies (Spiegel et al. 2016). In other systems, insects sequester macromolecules from their diet to then create a pheromone blend, as seen in tephritid flies (Shelly 2001; Segura et al. 2018), *Utetheseia* moths (Eisner and Meinwald 1995; Del Campo et al. 2005), and bark beetles (Byers 1983). There are only a few cases in which male insects collect and emit signals with exogenous compounds. Interestingly, those cases all involve bees: Males of certain species of *Paratetrapedia* (Etl et al. 2017), *Tetrapedia* (Cappellari et al. 2012) and *Anthophora* (Nordén and Batra 1985) collect volatiles thought to be involved in courtship. The best-known case involves all species of orchid bees (Euglossini) (Vogel 1966; Dressler 1982) in which male bees use specialized cuticular structures for the uptake of volatiles from their environment that are used in courtship display (Henske et al. 2023).

Male euglossine bees, or orchid bees (Apidae: Euglossini), collect odorants from environmental sources such as flowers, decaying wood, and feces, and even artificial sources like pesticides (Vogel 1966; Dressler 1968, 1982; Roberts et al. 1982; Whitten et al. 1993; Roubik and Hanson 2004; Ramírez et al. 2010; Henske et al. 2025). Male bees extract odorants through labial gland lipid secretions, a process similar to enfleurage in perfumery, and store them in modified hind tibial pockets (Vogel 1966; Eltz et al. 2007). Males use the crafted perfume in courtship display, forming a species-specific chemical signal that functions as a sex pheromone (Eltz et al. 1999, 2005; Zimmermann et al. 2009a; Henske et al. 2023). For display, male orchid bees find a perch (*eg*, a stem or branch) to land on and perform repeated hovering flights or wing buzzes. They perform characteristic leg movements that result in the exposure of the perfume and its dispersal downwind (Bembé 2004; Eltz et al. 2005; Pokorny et al. 2017). Individual males have been observed to display at the same or different perches for days or weeks, both in the wild and in cages (Kimsey 1980; Stern 1991; Henske et al. 2023). Females detect the airborne perfume, and then approach displaying males from downwind, for mating (Eltz et al. 2003; Henske et al. 2023). Multiple studies have shown that male orchid bee perfume composition varies more between species than within species (Zimmermann et al. 2009a; Weber et al. 2016). Furthermore, a species' unique chemical signal remains consistent across its entire geographic range, across regions with variable species occurring in sympatry (Zimmermann et al. 2009a; Ramírez et al. 2010; Weber et al. 2016; Darragh et al. 2023). Recent experimental evidence demonstrates that female *Euglossa dilemma* orchid bees prefer to mate with males that have been supplemented with conspecific perfume loads and avoid males that lack a perfume load (Henske et al. 2023). However, the exact quality and range of information conveyed by these environmentally acquired signals has yet to be experimentally tested.

Here, we ask whether perfume mixtures mediate species recognition among closely related sibling species. To answer this question, we turned to a pair of recently diverged sympatric species (∼0.15–0.11 mya), *E. dilemma* and *E. viridissima* (Eltz et al. 2011). The primary difference of perfume phenotypes in these species lies in the presence of different exclusive major compounds. *E. dilemma* perfumes are distinct by containing 4 stereoisomers of 2-hydroxy-6-nona-1,3-dienylbenzaldehyde (HNDB), whereas *E. viridissima* perfumes are dominated by an exclusive lactone derivative of linolenic acid (L97) (Eltz et al. 2008; Pokorny et al. 2013; Brand et al. 2020). The environmental sources of both HNDB and L97 are unknown in the natural habitat of *E. dilemma* and *E. viridissima*, but the clear partitioning of the compounds demonstrates that they are derived from different sources; HNDB, also referred to as albiducin B, has been synthesized and separately isolated from a European ash tree saprotrophic fungus, hinting at a potential fungal origin (Halecker et al. 2018; Stell et al. 2025). However, besides the exclusive major compounds, there is a broad overlap in the composition of the remaining perfume components found in both species. The distinctness in major compounds has been related to differential tuning of one odorant receptor, OR41, which mediates detection of HNDB in *Euglossa dilemma* (Brand et al. 2020). This differential tuning may have facilitated the rapid evolution of reproductive barriers, a process likely further enhanced by the fact that both signal production (*ie*, via male odorant collection) and signal perception (*ie*, via female response) are influenced by the same odorant receptor (Eltz et al. 2008; Brand et al. 2020). Here, we test the role of perfumes in species-assortative mating, which we achieved by first collecting perfume from wild males of the 2 species in areas of sympatry in Mexico that we then used to manipulate the perfume loads of perfume-free males of *Euglossa dilemma* in standardized cage mating experiments with perfume-naive females.

## Materials and methods

### Perfume collection and analysis

We collected perfume in regions of Mexico where *E. dilemma* and *E. viridissima* occur in sympatry, including the Yucatán Peninsula, Campeche, and the Los Tuxtlas region of Veracruz (QGIS Map and geographic coordinates; Fig. 1 and Table S1). We specifically selected sympatric regions to ensure that differences in perfume chemistry are the result of species divergences instead of habitat quality or availability of perfume compounds. The perfume collection took place at 6 sites in early June and October 2023 and 2024. Male bees were attracted using chemical baits, consisting of filter papers infused with synthetic volatiles enclosed in tea sieves; males are attracted to baits as they forage for perfume compounds, and certain compounds (*eg*, 1,4-dimethoxybenzene, eugenol) attract both species. As is generally the case in orchid bees, females rarely, if ever, approach single-compound baits. After netting the males, we used hand lenses to noninvasively distinguish between the 2 species using a key morphological trait (*ie*, mandibular teeth) (Eltz et al. 2008; Brand et al. 2020). To extract perfume, we squeezed the hind tibial pouch while pressing 2 µL end-to-end glass capillary tubes (Hirschmann Laborgeräte, Eberstadt, Germany) on the hind tibial slit to collect the extruding perfume (Fig. 2a); after extracting perfume, we then released each male bee. Perfumes were pooled from 5 to 7 sampled individuals into a 2 mL Agilent autosampler glass vial fitted with a 100 µL insert. We did not combine perfume pools from different locations.

**Figure 1:**
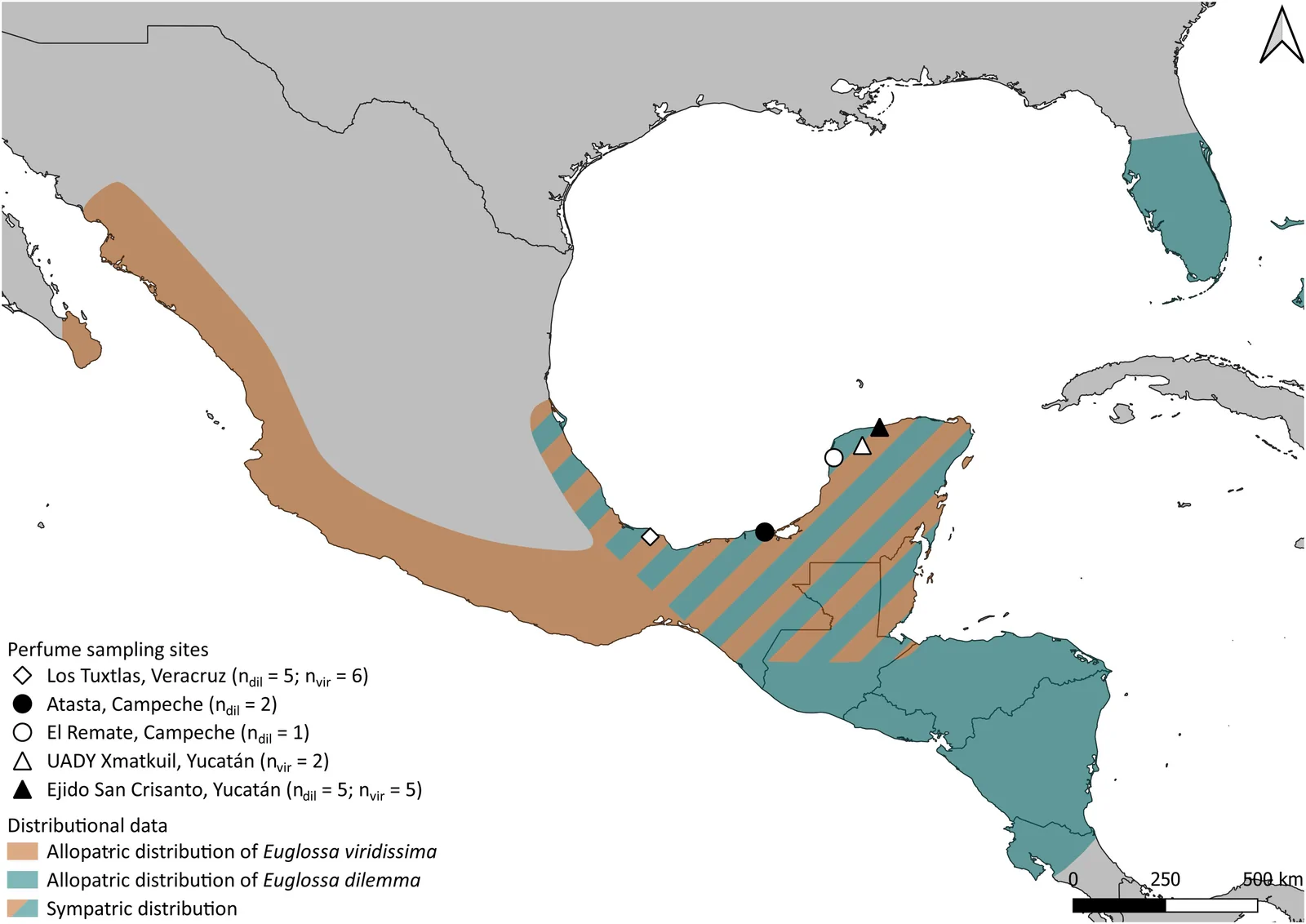
Geographic ranges of *Euglossa viridissima* and *Euglossa dilemma* (Eltz et al. 2011; Falcón Brindis et al. 2018; Pemberton and Escalona 2023) and localities where perfume was collected from males to create experimental perfume pools (Map created with QGIS version 3.44.8-Solothurn).

**Figure 2:**
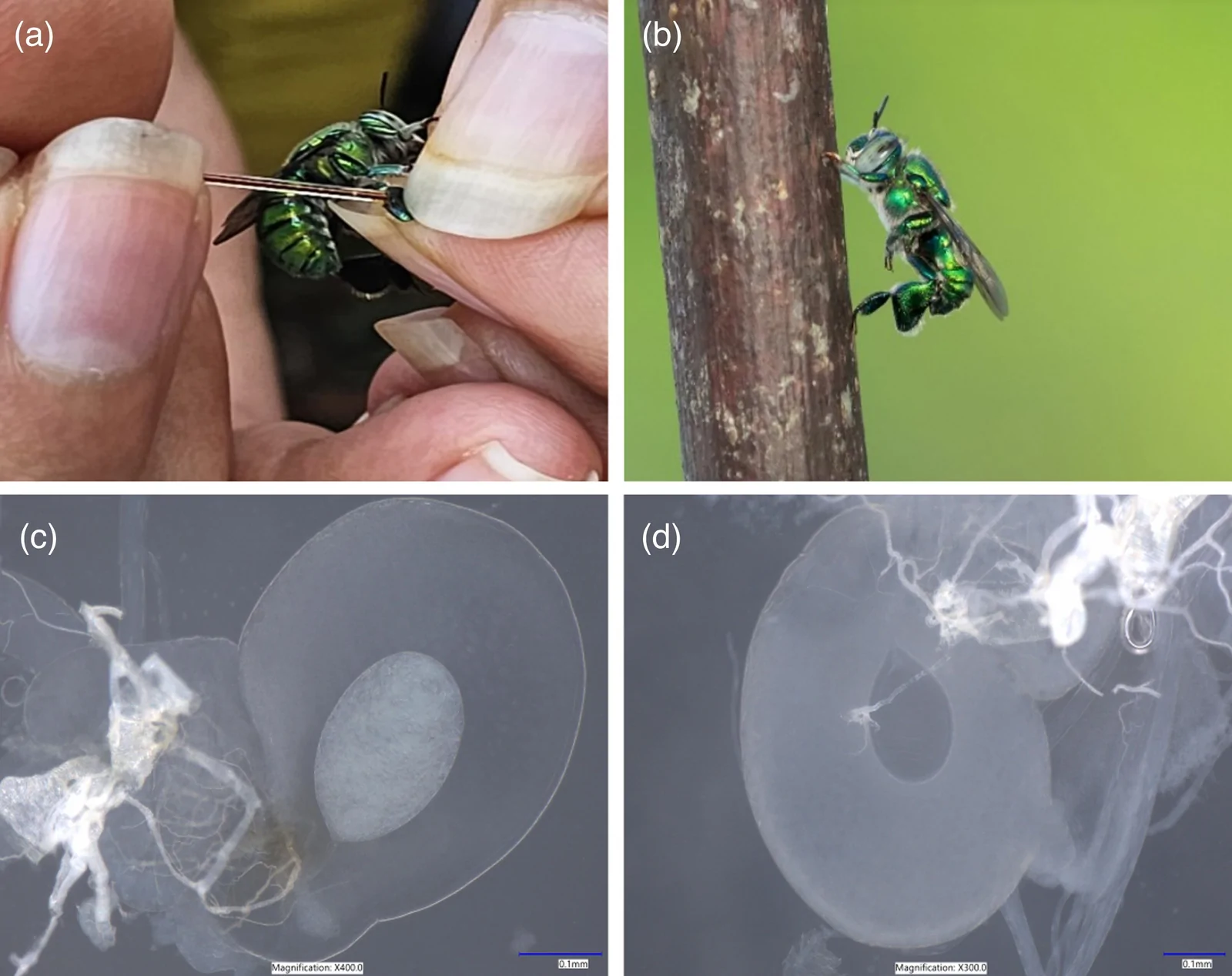
a) Perfume extraction from the hind leg of a male orchid bee using glass microcapillaries. Image by J. Dohrs. b) Characteristic courtship posture of a male orchid bee displaying on Senna spp. perch. Image by T. Eltz. c) Spermatheca of a mated female. d) Spermatheca of an unmated female.

Species identification was corroborated with chemical analyses of the perfume samples. From each of the pooled perfumes, we removed an aliquot of 0.1 µL with a microliter syringe and diluted it in 150 µL of *n*-hexane for gas chromatography-mass spectrometry analysis. These analyses were conducted using an Agilent 7890b gas chromatograph connected to an Agilent 5977a Mass Selective Detector, with a nonpolar HP-5 Ultra Inert column (Agilent, 30 m × 250 μm, 0.25 μm). We injected 1 µL of each of the fractions via an Agilent ALS 7694 autosampler in the splitless mode with the split valve opened at 1.2 min (250 °C injector temperature). The oven temperature was programed from 55 °C, held for 3 min, and then increased to 300 °C by 10 °C/min; once at 300 °C, the temperature was maintained for 1 min and after increased by 15 °C/min until 315 °C was reached and subsequently held for 5 min. Helium was used as a carrier gas with a constant flow rate set to 0.7 mL/min. The chromatograms, mass spectra, and retention times were analyzed with the Agilent MSD ChemStation software (Version G1701DA Revision D.01.00). The peaks were identified and cross-referenced using a user mass-spectral library (Eltz, unpublished) and a commercially available spectral library (Adams 2007). Chromatograms, raw data files, and a list of perfume compound information can be found in the data repository.

### Single-choice mating assays

Following Henske et al. (2023), we first conducted dual-choice mating experiments in flight cages during the summer of 2023 to assess the relative preference of a female when simultaneously exposed to a male with *E. dilemma* perfume and another male with *E. viridissima* perfume. However, under these circumstances, the experimental males performed little to no courtship behavior as males often interrupted each other's display. So, presumably interested in the other treatment male's perfume, competition hindered courtship display (see Supplementary material). We therefore adjusted our experimental design and conducted parallel single-choice mating assays with 1 treatment male and 1 female bee at a time from August through November of 2024. Individual experimental bees were reared from trap nests placed in regional parks in Broward County, Florida (permit number: ES2023-006). All the bees used were newly emerged males with no prior experience in volatile collection. Likewise, all the females used in the experiments were newly emerged and had not previously mated. Because all experimental males and females were *E. dilemma* bees, this approach permitted us to isolate the olfactory component of the signal, allowing us to assess absolute preference for perfume type.

Individual males were introduced to a 10 × 10 m flight cage upon emergence at the University of Florida Ft. Lauderdale Research and Education Center in Davie, Florida (see Supplementary material for details on flight cage materials). We began observations of display behavior on the subsequent day after cage introduction. Once the males showed characteristic display behavior on a perch (Eltz et al. 2005; Henske et al. 2023) (Fig. 2b), we injected 0.5 µL of undiluted field-collected *E. dilemma* or *E. viridissima* perfume into each hind tibial pouch. We used a different perfume pool for each case, so no 2 males received the same treatment perfume. Afterwards, we introduced a newly emerged, perfume-naive female bee, so each individual female was presented with only one male. The pair was kept in the flight cage for 3 to 5 total days (5 when inclement weather prevented display), during which we conducted standardized observations of male display activity (Eltz et al. 2005) in the mornings (8 AM until 12 PM), when the bees are most active. Display consists of a male bee performing characteristic perching and hovering behaviors on/near a perch (*eg*, a *Senna* spp. stem). An observation interval lasted for 10 min, during which we scanned all perch sites for display 3 times. Seven to ten such intervals were completed on each experimental day, usually spaced evenly over the observation period but occasionally clustered in periods due to weather conditions (see Henske et al. 2023). From these observations, the individual display rate was calculated by dividing the number of scans with active display activity by the total number of scans per male. We cycled treatments through all 3 flight cages to mitigate any cage effects. We completed 13 replicates of the mating assays for each perfume treatment. At the end of each experimental trial, the male and female bees were cooled down to reduce adverse stimuli prior to being sacrificed through freezing (−20 °C) for downstream analyses (ASAB Ethical Committee/ABS Animal Care Committee 2024). The University of California, Davis does not require ethical approvals for research involving insects (UC Davis 2026).

#### Spermatheca dissections

Female *E. dilemma* only mate once, both in nature and in captivity (Zimmermann et al. 2009b; Henske et al. 2023). Post mating, the sperm is stored in the spermatheca, which allows us to assess the mating status by the presence or absence of sperm (Fig. 2c, d). We extracted female spermathecae under a Leica S6E stereo microscope and visualized contents on microscopic slides using a Keyence VHX-7000 Optical Microscope.

#### Hind leg perfume compound confirmation

After each experimental trial, we removed the male from the flight cage, removed its hind tibiae, and extracted the perfume compounds in 500 µL of *n*-hexane for gas chromatography-mass spectrometry analyses. Hind tibial samples were run and analyzed using the same methods and protocol indicated above.

#### Statistical analyses

To test the effects of perfume treatment on male display rate, we used a 2-tailed *t*-test in R version 4.3.0, with the function *t.test* in the *stats* package (R Core Team 2023). Although one individual in the *E. viridissima* perfume treatment was not found at the end of the 3-d mark in the flight cage, this replicate was retained in the analysis of display rate but was excluded for nMDS perfume visualization.

To test for the effects of perfume treatment on the proportion of males that had successfully mated, we ran a Yates modified 2-tailed chi-square test. We tested the independence of copulation with 2 levels of “yes” and “no”, and perfume type with the 2 levels “*E. dilemma* perfume” and “*E. viridissima* perfume”. The Yates chi-square test was performed in R version 4.3.0 using the *chisq.test* function from the *stats* package (R Core Team 2023).

To measure perfume divergence in the hind tibial extracts between the species, we conducted a nonmetric multidimensional scaling analysis (nMDS) (Bray–Curtis similarity matrix, lowest *k*-value with stress <0.2 at *k* = 3 using the function *metaMDS* in the R package *vegan* (Oksanen et al. 2026)). To investigate whether species' perfumes exhibited more dissimilarity between than within respective species, and which components contribute the most to this dissimilarity, we performed an ANOSIM test, followed up by a SIMPER analysis in the R package *vegan* (Oksanen et al. 2026). We visualized the perfume composition using the *ggplot* function and tested for differences in complexity (number of compounds) using a 2-tailed *t*-test with the function *t.test* from the *stats* package (R Core Team 2023).

In *post-hoc* exploratory analyses of the hind tibial extracts, we investigated the role of HNDB, perfume richness, and perfume total amount on the likelihood of mating *within* the *E. dilemma* treatment only. We ran 3 generalized linear models for a binary outcome. Further details on these analyses can be found in the Supplementary Materials.

## Results

### The rate of male courtship behavior did not differ between perfume treatments

Regardless of whether males were loaded with the conspecific *E. dilemma* perfume or the heterospecific *E. viridissima* perfume, the individual display rates were not significantly different (*t* = 1.3462, df = 23.496, *n*_dilemma_ = 13, *n*_viridissima_ = 13, *P* = 0.1911, *t*-test). The average display rate was 0.43 and 0.31 for the *E. dilemma* and *E. viridissima* treatment, respectively. Male bees in the *E. dilemma* perfume treatment exhibited both the highest and lowest amounts of display (Fig. 3a).

**Figure 3:**
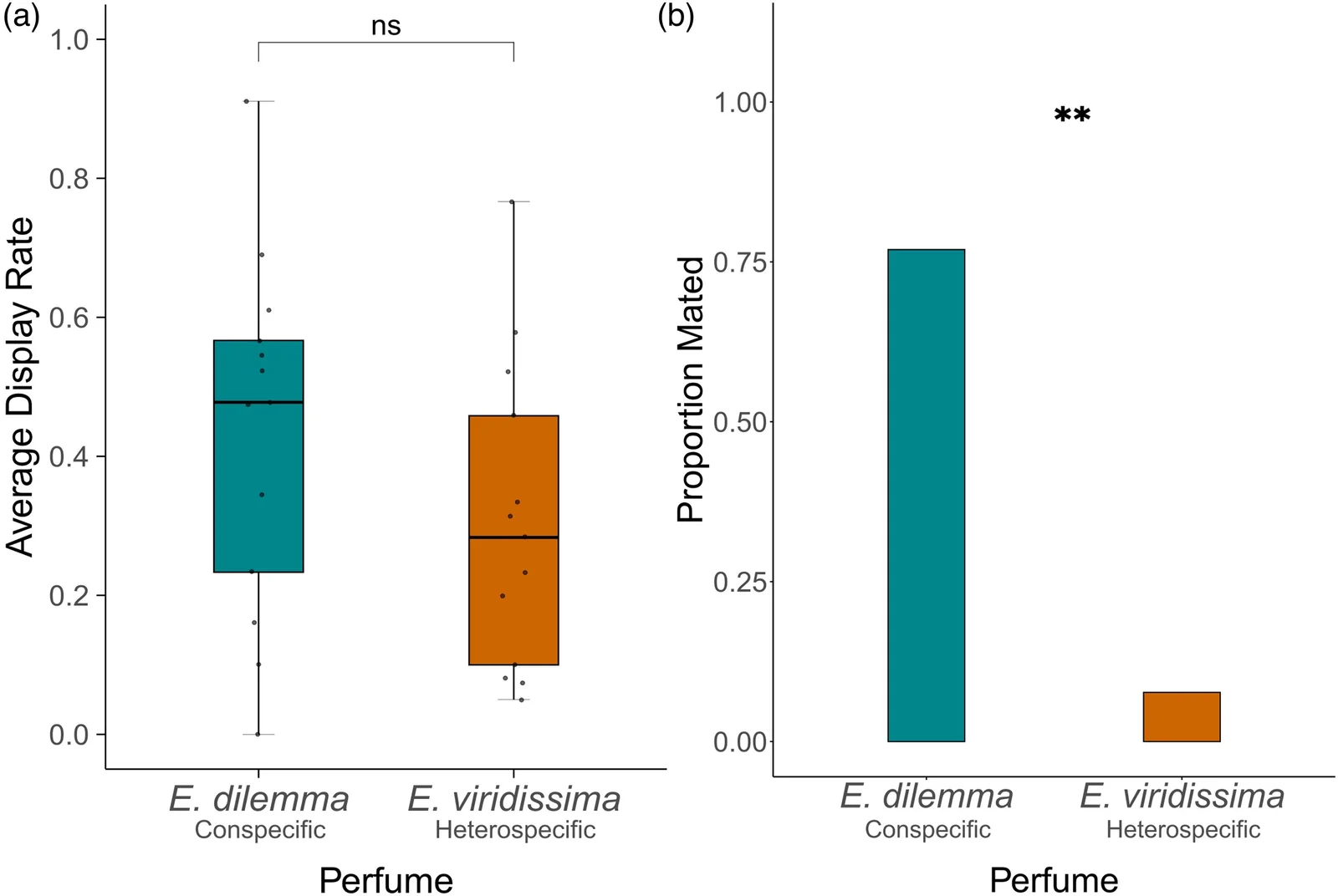
a) The display rate of *Euglossa dilemma* males supplemented with either conspecific (*E. dilemma*) or heterospecific (*E. viridissima*) perfume loads during single-choice mating assays. Display rate was calculated as the proportion of observational scans in which a male performed characteristic courtship behavior on a perch. No significant difference in display activity was detected between treatments (*t* = 1.3462, df = 23.496, *P* = 0.1911, *t*-test). b) The proportion of males mated across perfume treatments. Percentage of *E. dilemma* males that successfully mated when supplemented with either conspecific (*E. dilemma*) or heterospecific (*E. viridissima*) perfume blends during single-choice mating assays. Mating success was determined by the presence of sperm in the female spermatheca. Significantly more males supplemented with *E. dilemma* conspecific perfume mated (77%) compared with males loaded with *E. viridissima* heterospecific perfume (8%). (χ² (df = 1) = 10.09, **P = 0.0015, chi-squared test).

### Females prefer mates presenting conspecific perfume blends

Successful mating events were assessed through direct spermatheca dissections, with the presence of sperm scored as a mating and the absence scored as unmated. The proportion of mated male bees differed significantly between the perfume treatments, with 76.90% (10 out of 13 males) of the males mating in the *E. dilemma* perfume treatment and 7.69% (1 out of 13 males) in the *E. viridissima* perfume treatment (χ^2^ (df = 1) = 10.09, *P* = 0.0015, chi-squared test, Fig. 3b).

As shown in the nMDS plot, perfume compositions clustered distinctly by species along the first and second axes (stress = 0.14) (Fig. 4, ANOSIM *R* = 0.83, *P* = 0.0001). To explore the chemical basis underlying the multivariate separation, we examined the relative composition of the used perfumes in more detail. On average, about half of each species' perfume was made up by the respective major species-specific component, HNDB in its 4 stereoisomers in *E. dilemma*, and L97 in *E. viridissima*. The SIMPER analysis confirmed that L97 and HNDB were the top compounds that contributed to 56.8% of the overall between species chemical dissimilarity observed (Table S2). Besides these 2 major components, the perfumes of both species used in this experiment were mostly characterized by terpenoids (predominantly monoterpenes and sesquiterpenes) and aromatic compounds such as phenylpropanoids. Many of these compounds were found in both species but differed in their relative abundance (Fig. 5, Table S3). While there were differences in the mean number of components found in a species' perfume (8.38 components in *E. dilemma* and 5.50 in *E. virdissima*), these differences were not significant (*t*(*df* = 22.64) = 1.74, *P* = 0.096, *t*-test).

**Figure 4:**
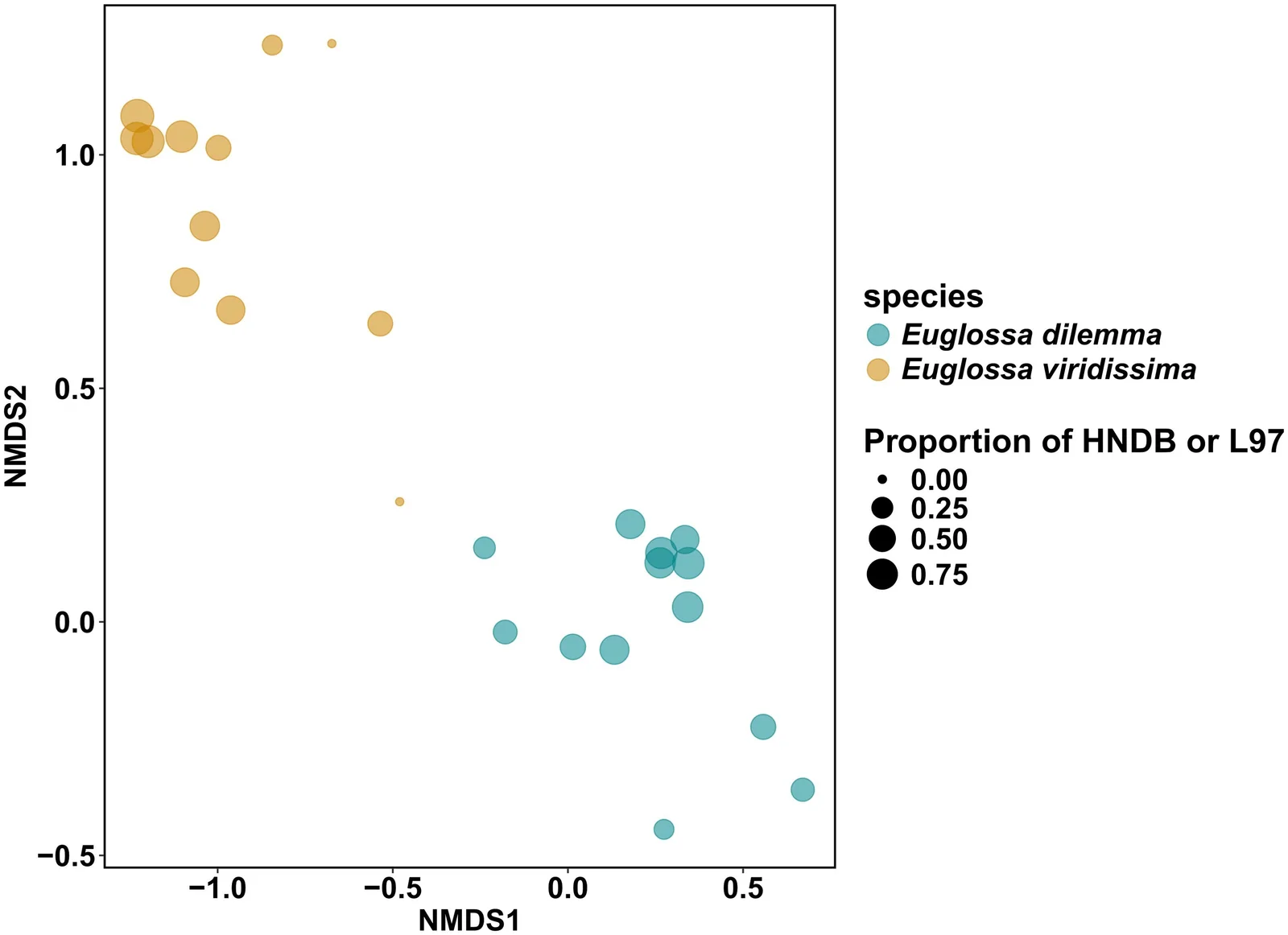
nMDS plot of sampled perfume for each species. The size of each point corresponds to the proportion of the main species-specific compounds, HNDB or L97, in the perfume. Perfumes were analyzed via GC/MS, a square matrix was generated to calculate Bray-Curtis distances, and the distances were used to generate a visual representation of perfume divergence.

**Figure 5:**
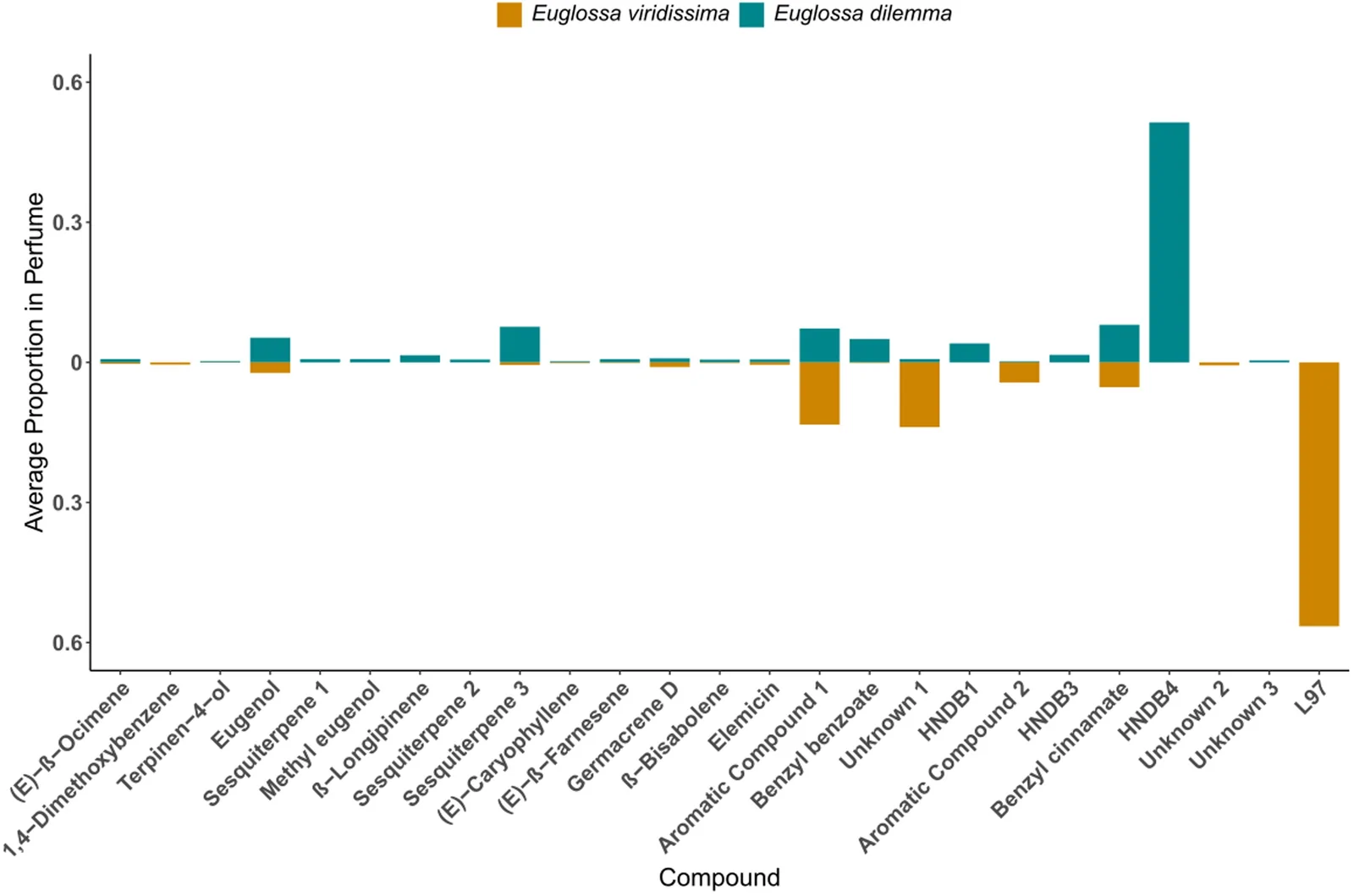
Average proportions of the 25 most abundant exogenous perfume components across species in the utilized perfume pools of *E. dilemma* and *E. viridissima* sorted from left to right by retention index, starting with the lowest. If possible, unknown compounds were characterized by their specific substance group (e.g., sesquiterpene), and the corresponding 10 most abundant fragment ions (m/z values) in the mass spectrum can be found in the Table S3.

Results for the *post-hoc* exploratory analyses and associated figures can be found in the Supplementary Materials (Figure S1 and Tables S4a–c); the analyses were limited by the small sample size, and all results were nonsignificant.

## Discussion

Our findings demonstrate a strong effect of perfume composition in mediating mate recognition in orchid bees. Our results revealed that females strongly prefer to mate with males supplemented with conspecific perfume (*E. dilemma*) than with males supplemented with heterospecific perfume (*E. viridissima*). We found that the majority of males in the *E. dilemma* perfume treatment successfully mated, while only one of the *E. viridissima* perfume treatment males mated.

These results suggest that perfume composition alone is a sufficient reproductive isolating mechanism under our experimental conditions. It is not uncommon for males of different species of orchid bees to be observed displaying in close proximity to one another in the field. For example, Pokorny et al. (2017) found 8 separate species displaying along a ridge trail in Costa Rica, providing ample opportunity for signal interference and/or hybrid matings. Although there are some differences in perch preferences among co-occurring species, these aspects may be insufficient to convey mate identity due to considerable overlap between species (Pokorny et al. 2017). In regions with a high species diversity in orchid bee communities, reaching up to 66 sympatric species (Padrón et al. 2018), differences in perfume signals among species may represent the main mechanisms that prevent hybrid mating. In combination with the finding that conspecific males respond to species-specific hind tibial extracts when presented at display sites (Zimmermann et al. 2006), our results further lend support to the hypothesis that perfume composition acts as the primary signal of mate recognition.

Our study provides insight into how female orchid bees respond to entire perfume phenotypes when selecting mates. Disentangling the behavioral valence of individual compounds in the mixture in mating assays will require careful experimental design and manipulations to determine which components elicit direct responses and which act as modifiers (Dougherty 2020). Interestingly, the only mating that occurred with a male in the *E. viridissima* treatment involved an injected *E. viridissima* perfume that lacked L97, the dominant compound present in most *E. viridissima* perfumes. This observation is intriguing and merits further exploration. We hypothesize that L97 modified the attraction of *E. dilemma* females to males treated with *E. viridissima* perfume in our experiments. Such compound-specific deterrence has already been invoked in creating a pattern of selective, highly specific attraction of male orchid bees to orchid floral fragrances (Williams and Dodson 1972). These authors found that adding even small amounts of odorants (*ie*, modifiers) to artificial floral scents that are not attractive to male bees of a given species will dramatically reduce the attractiveness of known attractants to males of that species. Similar mechanisms may mediate selective attraction to complex male tibial perfumes in females. In fact, adding specific compounds to individual perfumes may constitute a simple mechanism for establishing behavioral isolation among nascent species. The preference for HNDB may have evolved as a prezygotic barrier to prevent interbreeding with *E. viridissima* during incipient speciation.

Preference for chemical signals may have evolved through selection acting on both novel or mutually exclusive compounds, and sister taxa comparisons offer unique opportunities for future research for manipulative behavioral experiments. Consistent with reproductive isolation via reinforcement in other species (Noor 1995; Rundle and Schluter 1998; Higgie et al. 2000; Nosil et al. 2003; Wellenreuther et al. 2010; Pfennig and Rice 2014), sympatric orchid bee sister species exhibit more distinct perfumes than allopatric sister lineages (Weber et al. 2016). Furthermore, perfume traits in orchid bees evolve more rapidly than traits which are not involved in sexual signaling (Weber et al. 2016). In addition, Brand et al. (2015) showed that the genetic underpinnings of these phenotypes similarly evolve quickly, as expected, with divergent selection on male odorant receptors showing the highest rates of evolution out of the chemosensory genes. Because signal production and perception are the result of the same sensory system (olfactory genes), implicating rapid evolution (Rosenthal 2017), female preference likely evolves rapidly. We speculate that the rapid evolution of perfume traits is shaped by several factors, including selection against hybrid matings (*ie*, reinforcement) and/or sexual selection (*ie*, strong competition for mates); these factors need not be mutually exclusive, and the extent to which female preference plays a role in responding to minor and major perfume compounds remains unknown.

Because male orchid bees craft perfume in what could be called an extended phenotype, this trait has long captivated researchers due to the juxtaposition of a necessary sexual signal for mating in conjunction with an ephemeral resource to achieve it. Here, we present experimental evidence of female orchid bee preference for their conspecific perfume and their ability to discriminate between conspecific and heterospecific perfumes. In combination with previous research, these findings further corroborate the linkage between male odor collection behavior and female mating behavior, with recognition and subsequent choice mediated by perfume composition. Our results are congruent with a scenario of chemically driven mate recognition facilitating reproductive isolation.

## Supplementary Material

arag124_Supplementary_Data

## Data Availability

Analyses reported in this article can be reproduced using the data and R code provided by Sandoval et al. (2026).
