# Supplementary material for: Mate recognition in orchid bees is mediated by acquired chemical signals
Source: Behav Ecol. 2026 Oct 7;37(6):arag124. doi: 10.1093/beheco/arag124 (PMC13687648; doi:10.1093/beheco/arag124)
Supplement: Supplementary file 1 [file arag124_supplementary_data.zip › Supplementary materials and figures.docx]

**Supplementary materials and figures**

***Post-hoc* generalized linear models within the *E. dilemma* treatment**

**Methods:**

As in *post-hoc* exploratory analyses, we investigated the role of HNDB (*i.e*., the dominant exclusive perfume compound for *E. dilemma*), perfume richness, and perfume total amount on the likelihood of mating *within* the *E. dilemma* treatment only via three generalized linear models for a binary outcome. For each model, the continuous predictor was the proportion of HNDB in the perfume, perfume richness (number of perfume compounds), or perfume total amount (summed peak areas, *i.e.,* total integrated ion currents), and the response categorical variables were once again “mated” (1) or “unmated” (0). We predicted a positive relationship between the increase in these predictors and the increased likelihood of mating.

The variables investigated were obtained from the hind tibial extracts. We fitted each model using the function *glm* and specified the family as binomial with a logit link in R (R Core Team, 2023).

**Results and Discussion:**

From running the *E. dilemma* perfume GLMs, we found no significant effects of the proportion of HNDB, perfume complexity, or the total amount of perfume on the mating outcome (Suppl. Fig. 1a-c, p = 0.91, p = 0.92, and p = 0.17). Results are reported in the Supplementary Tables 4a-c with summary statistics from the model outputs. The strongest trend was found for the total amount of perfume (p = 0.17).

Overall, the total amount of perfume, a proxy of stimulus intensity, had the strongest trend in explaining successful mating. Even though HNDB is an outstanding perfume compound in *E. dilemma* in terms of abundance, our data lacked statistical power due to a low replicate size to determine how this HNDB alone affects the likelihood of mating. Manipulative experiments are required to disentangle the contribution of each compound to mate preferences. Our findings suggest that behavioral specificity and mate choice in orchid bees are shaped by the emergent properties of the complex specific-specific perfume blends rather than by any individual compound (Gamboa et al., 1986, 1991; Lacy & Sherman, 1983; Sherman et al., 1997; Waldman, 1987). This result mirrors results from other studies of signal recognition. For example, in nestmate recognition of social insects, it has been shown that no single compound in isolation from a pheromone blend explains behavioral responses, including aggression or acceptance of intruders and nestmates, respectively, but instead arises from the emergent property of the blend that triggers responses (Buchwald & Breed, 2005; Kaib et al., 2004; Reichle et al., 2013).

Supplementary Table 1. Perfume sampling coordinates for field sites in Mexico.

| Sampling locality | Latitude | Longitude |
| --- | --- | --- |
| Ejido San Crisanto, Yucatán | 21.3421246 | -89.1740275 |
| UADY Xmatkuil, Yucatán | 20.867883 | -89.624435 |
| Atasta, Campeche | 18.6113428 | -92.1609311 |
| El Remate, Campeche | 20.5347062 | -90.3699910 |
| San Martín Tuxtla, Veracruz | 18.5365484 | -95.138052 |
| Adolfo Ruiz Cortinez/Los Tuxtlas, Veracruz | 18.5530308 | -95.1539404 |

Supplementary Table 2. SIMPER analysis table. The compounds are ranked by the contribution to between-species chemical dissimilarity. Unknown components were, whenever possible, characterized by their ten most abundant fragment ions in the mass spectrum (m/z).

| Compound | Average contribution to species differences | sd | Cumulative Sum | p |
| --- | --- | --- | --- | --- |
| L97 | 0.282558 | 0.166356 | 0.297245 | 0.001 |
| HNDB4 | 0.256925 | 0.10635 | 0.567526 | 0.001 |
| aromatic compound (m/z: 208, 193, 77, 79, 104, 91, 133, 165, 135, 209) | 0.083044 | 0.132957 | 0.654887 | 0.478 |
| Unknown (m/z: 88, 76, 171, 41, 77, 227, 213, 39, 195, 104) | 0.070945 | 0.128252 | 0.729519 | 0.07 |
| Benzyl cinnamate | 0.055572 | 0.065463 | 0.78798 | 0.024 |
| Sesquiterpene (m/z: 120, 161, 91, 105, 41, 79, 77, 93, 119, 81) | 0.039186 | 0.066199 | 0.829203 | 0.187 |
| Eugenol | 0.02876 | 0.029038 | 0.859458 | 0.212 |
| Benzyl benzoate | 0.024673 | 0.022305 | 0.885413 | 0.001 |
| Aromatic compound (m/z: 225, 240, 226, 210, 182, 152, 76, 181, 165, 153) | 0.022242 | 0.038337 | 0.908812 | 0.034 |
| HNDB1 | 0.020276 | 0.016544 | 0.930141 | 0.001 |
| HNDB3 | 0.007955 | 0.007958 | 0.938509 | 0.007 |
| Germacrene D | 0.007809 | 0.011023 | 0.946725 | 0.347 |
| β-Longipinene | 0.00762 | 0.014474 | 0.954741 | 0.105 |
| (E)-β-Ocimene | 0.004301 | 0.007003 | 0.959265 | 0.898 |
| Elemicin | 0.004173 | 0.004325 | 0.963655 | 0.687 |
| (E)-β-Farnesene | 0.003704 | 0.006503 | 0.967551 | 0.502 |
| Methyl eugenol | 0.003424 | 0.011899 | 0.971153 | 0.487 |
| Sesquiterpene (m/z: 108, 91, 105, 93, 161, 40, 79, 133, 119, 77) | 0.00337 | 0.008024 | 0.974698 | 0.236 |
| β-Bisabolene | 0.003359 | 0.007163 | 0.978232 | 0.732 |
| Unknown | 0.003219 | 0.00754 | 0.981618 | 0.001 |
| Sesquiterpene (m/z: 91, 105, 161, 41, 93, 94, 107, 77, 79, 119) | 0.00303 | 0.005788 | 0.984806 | 0.105 |
| 1,4-Dimethoxybenzene | 0.002331 | 0.003696 | 0.987258 | 0.001 |
| Unknown (m/z: 88, 101, 43, 55, 41, 69, 57, 89, 70, 71) | 0.002057 | 0.007148 | 0.989421 | 0.505 |
| (E)-Caryophyllene | 0.001924 | 0.004398 | 0.991445 | 0.749 |
| Terpinen-4-ol | 0.001496 | 0.003157 | 0.993019 | 0.448 |
| Unknown (m/z: 165, 91, 138, 106, 77, 65, 272, 92, 78, 150) | 0.001319 | 0.004584 | 0.994407 | 0.487 |
| α-Farnesene | 0.001038 | 0.003609 | 0.995499 | 0.482 |
| Unknown (m/z: 41, 78, 43, 69, 93, 91, 105, 107, 54, 77) | 0.00085 | 0.00219 | 0.996394 | 0.001 |
| Unknown (m/z: 57, 43, 41, 54, 55, 96, 68, 82, 44, 41) | 0.00065 | 0.002259 | 0.997077 | 0.482 |
| Unknown (m/z: 40, 69, 109, 43, 82, 67, 39, 55, 84, 53) | 0.000556 | 0.00185 | 0.997662 | 0.001 |
| (−)-β-Elemene | 0.000551 | 0.001914 | 0.998242 | 0.478 |
| HNDB2 | 0.000451 | 0.001567 | 0.998716 | 0.487 |
| β-Gurjunene | 0.00045 | 0.001563 | 0.999189 | 0.478 |
| Unknown (m/z: 256, 31, 199, 128, 72, 245, 54, 152, 56, 79) | 0.000361 | 0.001202 | 0.999569 | 0.001 |
| Unknown (m/z: 55, 67, 43, 97, 83, 69, 130, 56, 90, 67) | 0.000212 | 0.000735 | 0.999792 | 0.487 |
| Unknown (m/z: 81, 79, 92, 121, 104, 68, 67, 41, 95, 54) | 0.000198 | 0.000687 | 1 | 0.487 |

Supplementary Table 3. Top 25 most abundant exogenous compounds across species in the utilized perfumes of *E. dilemma* and *E. viridissima* sorted by their Kovats retention indices. Unknown components were, whenever possible, characterized by their ten most abundant fragment ions in the mass spectrum (m/z) and a substance class.

| Substance Name | Substance Class | Kovats Retention Index |
| --- | --- | --- |
| (E)-β-Ocimene | Monoterpene | 1064 |
| 1,4-Dimethoxybenzene | Aromatic compund | 1170 |
| Terpinen-4-ol | Monoterpene alcohol | 1185 |
| Eugenol | Phenylpropanoid | 1365 |
| Sesquiterpene 1 (m/z: 108, 91, 105, 93, 161, 40, 79, 133, 119, 77) | Sesquiterpene | 1411 |
| Methyl eugenol | Phenylpropanoid | 1413 |
| β-Longipinene | Sesquiterpene | 1421 |
| Sesquiterpene 2 (m/z: 91, 105, 161, 41, 93, 94, 107, 77, 79, 119) | Sesquiterpene | 1430 |
| Sesquiterpene 3 (m/z: 120, 161, 91, 105, 41, 79, 77, 93, 119, 81) | Sesquiterpene | 1440 |
| (E)-Caryophyllene | Sesquiterpene | 1442 |
| (E)-β-Farnesene | Sesquiterpene | 1469 |
| Germacrene D | Sesquiterpene | 1503 |
| β-Bisabolene | Sesquiterpene | 1525 |
| Elemicin | Phenylpropanoid | 1559 |
| Aromatic compound 1 (m/z: 208, 193, 77, 79, 104, 91, 133, 165, 135, 209) | Phenylpropanoid? | 1655 |
| Benzyl benzoate | Aromatic compund | 1782 |
| Unknown 1 (m/z: 88, 76, 171, 41, 77, 227, 213, 39, 195, 104) | Unknown | 1950 |
| HNDB1 | Aromatic compund | 1967 |
| Aromatic compound 2 (m/z: 225, 240, 226, 210, 182, 152, 76, 181, 165, 153) | Aromatic compund | 1988 |
| HNDB3 | Aromatic compund | 2074 |
| Benzyl cinnamate | Aromatic compund | 2109 |
| HNDB4 | Aromatic compund | 2134 |
| Unknown 2 | Unknown | 2180 |
| Unknown 3 (m/z: 88, 101, 43, 55, 41, 69, 57, 89, 70, 71) | Unknown | 2203 |
| L97 | Fatty acid derivative | 2310 |

Supplementary Tables 4a-c. Results of generalized linear model on the effect of a). the proportion of HNDB, b.) perfume richness, and c.) perfume total amount on mating outcomes.

4a.)

| Explanatory variable | Estimate | SE | *Z* | *p* |
| --- | --- | --- | --- | --- |
| Intercept | 2.434 | 2.272 | 1.072 | 0.284 |
| Proportion of HNDB | -1.938 | 3.296 | -0.588 | 0.557 |

4b.)

| Explanatory variable | Estimate | SE | *Z* | *p* |
| --- | --- | --- | --- | --- |
| Intercept | 0.9863 | 1.4045 | 0.702 | 0.483 |
| Perfume richness | 0.0264 | 0.1529 | 0.173 | 0.863 |

4c.)

| Explanatory variable | Estimate | SE | *Z* | *p* |
| --- | --- | --- | --- | --- |
| Intercept | 3.660e-01 | 1.110e+00 | 0.330 | 0.742 |
| Perfume total amount | 6.351e-08 | 7.713e-08 | 0.823 | 0.410 |

**Dual-choice mating assay in 2023**

**Methods:**

We performed the dual-choice mating assays in 10 × 10 m flight cages from August through November of 2023 at the University of Florida Ft. Lauderdale Research and Education Center in Davie, Florida. The cages contained several *Pentas lanceolata* and *Stachytarpheta* spp. as nectar sources, and *Senna* spp. were offered as pollen sources. For brood cell construction, stingless bees’ wax was placed on vertical structures inside the cages. Individual experimental bees were procured from trap nests placed in regional parks in Broward County, Florida (permit number: ES2021-003). All the bees used were newly emerged males with no volatile collection experience and females had never mated.

Two newly emerged *E. dilemma* males were introduced to a flight cage at the same time, and once they both exhibited characteristic courtship behavior, 0.5 µL of either *E. dilemma* or *E. viridissima* perfume was injected into each hind tibial pouch of each respective treatment male. Each experimental replicate consisted of one male injected with *E. dilemma* perfume and one male injected with *E. viridissima* perfume. Males were marked with a scratch on the thorax to identify treatment, and females with a glued Opalith numbered tag on the thorax. We had seven experimental males per perfume treatment (n = 14 males). Then, newly emerged females were introduced to the cage. Observations of courtship behavior were conducted for five days after female introduction. We began observations of display activity from 8 AM until 12 PM, with an observational interval lasting 10 minutes. We scanned perch sites (e.g., *Senna* stems) in a standard way, three times per 10-minute interval. Ten intervals of observations were recorded per day, with occasionally fewer due to weather conditions. The individual display rate was calculated by dividing the number of observations with active display activity by the total number of observations. Observations were only counted if females were present, and both males were supplemented with their respective perfume treatment. At the end of each experimental trial, the male and female bees were cooled down to reduce adverse stimuli prior to being sacrificed through freezing (-20 °C) for downstream analyses (ASAB Ethical Committee/ABS Animal Care Committee, 2024).

**Results:**

**Males performed significantly less courtship behavior when there were two perfume-bearing males present in the cage**

All male bees performed little to no courtship behavior when they were paired with another perfume-bearing male (n = 14, 7 per perfume treatment). There was no significant difference in the individual display rate between perfume treatments in 2023, with males supplemented with *E. dilemma* exhibiting an average display rate of 0.10 and males supplemented with *E. viridissima* exhibiting an average display rate of 0.09 (W = 23.5, p = 0.9482, Wilcoxon rank-sum test). Instead of courtship display behavior, we observed far more interactions between the two males at and around perch sites (Suppl Fig. 2 and 3).

**References**

ASAB Ethical Committee/ABS Animal Care Committee. (2024). Guidelines for the ethical treatment of nonhuman animals in behavioural research and teaching. *Animal Behaviour*, *207*, I–XI. https://doi.org/10.1016/S0003-3472(23)00317-2

Buchwald, R., & Breed, M. D. (2005). Nestmate recognition cues in a stingless bee, *Trigona fulviventris*. *Animal Behaviour*, *70*(6), 1331–1337. https://doi.org/10.1016/j.anbehav.2005.03.017

Gamboa, G. J., Reeve, H. K., Ferguson, I. D., & Wacker, T. L. (1986). Nestmate recognition in social wasps: The origin and acquisition of recognition odours. *Animal Behaviour*, *34*(3), 685–695. https://doi.org/10.1016/S0003-3472(86)80053-7

Gamboa, G. J., Reeve, H. K., & Holmes, W. G. (1991). Conceptual Issues and Methodology in Kin‐recognition Research: A Critical Discussion. *Ethology*, *88*(2), 109–127. https://doi.org/10.1111/j.1439-0310.1991.tb00267.x

Kaib, M., Imhasly, P., Wilfert, L., Durka, W., Franke, S., Francke, W., Leuthold, R. H., & Brandl, R. (2004). Cuticular Hydrocarbons and Aggression in the Termite *Macrotermes Subhyalinus*. *Journal of Chemical Ecology*, *30*(2), 365–385. https://doi.org/10.1023/B:JOEC.0000017983.89279.c5

Lacy, R. C., & Sherman, P. W. (1983). Kin Recognition by Phenotype Matching. *The American Naturalist*, *121*(4), 489–512. JSTOR.

R Core Team. (2023). *R: A language and environment for statistical computing.* [Computer software]. https://www.R-project.org/

Reichle, C., Aguilar, I., Ayasse, M., Twele, R., Francke, W., & Jarau, S. (2013). Learnt information in species-specific ‘trail pheromone’ communication in stingless bees. *Animal Behaviour*, *85*(1), 225–232. https://doi.org/10.1016/j.anbehav.2012.10.029

Sherman, P. W., Reeve, H. K., & Pfennig, D. W. (1997). Recognition systems: An evolutionary approach. In N. B. Davies & J. R. Krebs (Eds.), *Behavioural ecology: An evolutionary approach* (4th ed). Blackwell Science.

Waldman, B. (1987). Mechanisms of kin recognition. *Journal of Theoretical Biology*, *128*(2), 159–185. http s://doi.org/10.1016/S0022-5193(87)80167-4
